# Low-Concentration Oxygen/Ozone Treatment Attenuated Radiculitis and Mechanical Allodynia via PDE2A-cAMP/cGMP-NF-*κ*B/p65 Signaling in Chronic Radiculitis Rats

**DOI:** 10.1155/2018/5192814

**Published:** 2018-12-13

**Authors:** Junnan Wang, Mingyi Wu, Xiaowen Lin, Yun Li, Zhijian Fu

**Affiliations:** ^1^Department of Pain Management, Shandong Provincial Hospital Affiliated to Shandong University, 324 Jingwu Road, Jinan, Shandong 250021, China; ^2^Department of Anesthesiology, Shandong Provincial Corps Hospital of Chinese People's Armed Police Forces, Jinan, Shandong 250014, China

## Abstract

**Background:**

Oxygen/ozone therapy is a minimally invasive technique for the treatment of radiculitis from lumbar disc herniation. This study aimed at investigating whether intrathecal administration of low-concentration oxygen/ozone could attenuate chronic radiculitis and mechanical allodynia after noncompressive lumbar disc herniation and at elucidating the underlying mechanisms.

**Methods:**

First, we transplanted autologous nucleus pulposus into dorsal root ganglions to establish chronic radiculitis in rats. Then, filtered oxygen or oxygen/ozone (10, 20, or 30 *μ*g/mL) was intrathecally injected on day 1 after surgery. The ipsilateral paw withdrawal thresholds (PWTs) to mechanical stimuli were tested daily with von Frey filaments. The expression of the tumor necrosis factor- (TNF-) *α*, interleukin- (IL-) 1*β*, IL-6, cyclic adenosine monophosphate (cAMP), cyclic guanosine monophosphate (cGMP), phosphodiesterase 2A (PDE2A), and nuclear factor- (NF-) *κ*B/p65 in spinal dorsal horns was measured by enzyme-linked immunosorbent assay, polymerase chain reaction, and western blot on day 7 after surgery.

**Results:**

Chronic radiculitis was established in rats. Intrathecal administration of 10 *μ*g/mL, 20 *μ*g/mL, or 30 *μ*g/mL oxygen/ozone significantly attenuated the decreased mechanical PWTs, downregulated the overexpression of spinal TNF-*α*, IL-1*β*, and IL-6, and increased the expression of cGMP and cAMP in chronic radiculitis rats. In addition, the effects of treatment with 20 *μ*g/mL oxygen/ozone were greater than the effects of the 10 *μ*g/mL or 30 *μ*g/mL doses. Moreover, intrathecal administration of 20 *μ*g/mL oxygen/ozone reversed the increased levels of spinal PDE2A and NF-*κ*B/p65 mRNA and protein expressions in rats with chronic radiculitis.

**Conclusion:**

Intrathecal administration of low-concentration oxygen/ozone alleviated mechanical allodynia and attenuated radiculitis, likely by a PDE2A-cGMP/cAMP-NF-*κ*B/p65 signaling pathway in chronic radiculitis rats.

## 1. Introduction

Radiculitis induced by lumbar disc herniation (LDH) is a very widespread and disturbing disease all over the world. A great deal of research has focused on LDH, and it has been clearly proven that radicular inflammation plays a key role in radicular pain in LDH patients [1–4]. Administration of epidural glucocorticoids and local anesthetics is a common treatment for radiculitis in pain management [5, 6]. However, the use of epidural glucocorticoids is limited by adverse effects. In the past decade, O_3_ therapy has gradually become one of the minimally invasive treatments available for radiculitis [7, 8]. A study by Melchionda et al. found that paravertebral oxygen/ozone (O_3_) therapy was safe and effective in patients with lumbar radiculitis compared to nonsteroidal anti-inflammatory drugs (NSAIDs) [9, 10]. O_3_ therapy is deemed to be an effective minimally invasive therapeutic strategy for LDH, radiculitis, and other chronic painful disorders.

However, ozone is a controversial gas. In previous studies, many researchers have found that ozone is toxic to some organs. Additionally, chronic inhalation of ozone upregulates proinflammatory cytokines in the respiratory system [11]. Some professors thought that ozone might always be toxic due to worsening of chronic oxidative stress, while on the contrary, others thought that the proper concentration of ozone could be atoxic and activate antioxidative systems [12, 13]. In previous studies, Li et al. found that 40 *μ*g/mL or 60 *μ*g/mL O_3_ treatments are neurotoxic to spinal neurons, whereas 20 *μ*g/mL or 30 *μ*g/mL O_3_ treatment decreased the levels of methane dicarboxylic aldehyde (MDA) and TNF-ɑ [14]. Carlo demonstrated that low concentration of O_3_ prevented the development of mechanical allodynia and decreased the overexpression of caspase 1, 8, and 12 in the orbitofrontal cortex in spared nerve injury (SNI) mice [15]. However, the underlying mechanisms of O_3_ therapy in radiculitis are not yet clear [12, 13, 16]. The current study aimed at exploring the mechanisms of a single intrathecal administration of low-concentration O_3_ in the rat with chronic radiculitis.

Phosphodiesterases (PDEs) are the only enzymes that hydrolyze cyclic adenosine monophosphate (cAMP) and/or cyclic guanosine monophosphate (cGMP) and are divided into 11 families (PDE1 to PDE11). Recent studies have demonstrated that inhibitors of PDEs inhibit the release of proinflammatory cytokines by cAMP/cGMP signaling pathways in depression, pulmonary hypertension, erectile dysfunction, and sepsis [17, 18]. Wiebke and Ruirui et al. found transient PDE2A mRNA upregulation after hind paw inflammation that was accompanied by enhanced acute mechanical paw withdrawal latencies, which indicated that PDE2A likely contributed to acute radicular inflammation and pain [19]. PDE2A can hydrolyze both cAMP and cGMP and preferentially hydrolyzes cAMP after activation by cGMP [20]. In our previous study, we found that PDE2A was significantly increased in the spinal cord of chronic radiculitis rats and was dramatically inhibited by the PDE2A inhibitor, BAY 60-7550, which decreased the spinal tumor necrosis factor- (TNF-) *α*, interleukin- (IL-) 1*β*, and IL-6 levels and also alleviated radiculitis and mechanical allodynia in noncompressive lumbar disc herniation (NCLDH) rats [21]. However, the relationship between PDE2A and O_3_ therapy has not yet been thoroughly studied.

In the present study, we investigated whether intrathecal injection of low-concentration O_3_ improved radicular pain in chronic radiculitis rats. Furthermore, we gained insights into whether PDE2A is involved in the underlying mechanisms of the treatment of pain by low-concentration O_3_ therapy.

## 2. Materials and Methods

### 2.1. Animals

Adult male Wistar rats (250–350 g) were purchased from the Experimental Animal Center of Shandong University in Shandong, China. All rats were housed in controlled environmental conditions (12 h light/12 h dark cycles and room temperature at 20–22°C) for 1 week prior to the experiment with ad libitum access to food and water. The experimental procedures were approved by the Institutional Animal Care and Use Committee of Shandong University. All endeavors were made to minimize animal suffering and the number of animals.

The investigator was blinded, and the rats were randomized into five groups (*n* = 8 per group), as follows:

- Sham group: sham operation, with a single intrathecal administration of filtered air
- Control group: NCLDH rats, with a single intrathecal administration of filtered air
- O_3_ 10 *μ*g/mL group: NCLDH rats, with a single intrathecal administration of 10 *μ*g/mL O_3_
- O_3_ 20 *μ*g/mL group: NCLDH rats, with a single intrathecal administration of 20 *μ*g/mL O_3_
- O_3_ 30 *μ*g/mL group: NCLDH rats, with a single intrathecal administration of 30 *μ*g/mL O_3_

### 2.2. Establishment of NCLDH Rats and Intrathecal Catheter Implantation

These procedures were completed as previously described [1, 21]. Rats were anesthetized with 10% chloral hydrate (3.0–3.5 mL/kg, i.p.) and fixed in the prone position. Using sterile procedures, right L5 vertebral laminae were exposed by a longitudinal incision at the midline. The right L5 dorsal root ganglion (DRG) and nerve root were exposed after cutting the right L5 vertebral laminae. The epineurium of the L5 DRG was then punctured to create a 2-3 mm opening. Following this, approximately 0.5 mg of the tail nucleus pulposus (NP) was obtained from between two coccygeal intervertebral discs and placed on the right L5 DRG, without mechanical compression. After that, a PE-10 cannula was inserted in the right L5 intervertebral foramina into the intrathecal space, approximately 0.5 cm caudal to the head. Cerebrospinal fluid flowed out of the cannula after placement. Proper intrathecal cannula placement was confirmed by the bilateral hind limb dragging or weakness after intrathecal administration of 10 *μ*L of 2% lidocaine [2, 22]. The catheter was fixed and sealed.

In the sham group, a right L5 hemilaminectomy was performed and then an intrathecal catheter was placed, without the implantation of NP or damage to the DRG.

### 2.3. O_3_ Administration

Filtered air or O_3_ was injected 24 h after surgery after evaluating mechanical paw withdrawal thresholds (PWTs). A single dose of filtered air (20 *μ*L) or O_3_ (10, 20, or 30 *μ*g/mL O_3_, 20 *μ*L) was administered over a period of 20 s by the microsyringe. O_3_ mixture was created by a medical ozone generator (CHY-31, Yuehua Company, China). The concentration was measured by the concentration detector on the medical ozone generator.

### 2.4. Mechanical Paw Withdrawal Thresholds (PWTs)

It has been confirmed that NCLDH rats have significant ipsilateral mechanical allodynia, but not thermal hyperalgesia [21–23]. Based on these previous findings we only evaluated ipsilateral mechanical paw withdrawal thresholds (PWTs) before surgery (day 0) and daily for 7 days after the operation (days 1–7). The mechanical PWTs in the right hind paw were measured by an experimenter blinded to treatment groups using von Frey monofilaments (0.41, 0.70, 1.20, 2.04, 3.63, 5.50, 8.51, and 15.14 g; Stoelting, Wood Dale, IL, USA). Ipsilateral mechanical PWTs were tested by the “up-down” method. A sudden withdrawal of the right hind paw after stimulation was seen as a positive response. The responses were then converted to a 50% threshold (50% threshold = 10^(*X*−*κd*)^/10^4^) as previously published [20].

### 2.5. Spinal Specimen

All animals were euthanized after PWTs were tested on day 7 after the operation. Rats were anesthetized with 10% chloral hydrate and then received cardiac perfusion with phosphate-buffered saline (PBS). The right spinal dorsal horns from L4–L6 were exposed, rapidly separated, and then stored in a −80°C freezer.

### 2.6. Enzyme-Linked Immunosorbent Assay (ELISA)

TNF-*α*, IL-1*β*, IL-6, cAMP, and cGMP levels in the spinal cord were measured by ELISA. The tissues were homogenized in PBS solution (pH 7.4, containing 1% Triton-X100, 1 mM PMSF, 10 g/mL aprotinin, and 1 g/mL leupeptin). The homogenized samples were subsequently centrifuged at 10000 g for 30 minutes at 4°C. Then, the supernatant was aliquoted and stored at −80°C. Supernatants were assayed using the manufacturer's instructions for the rat TNF-*α*, IL-1*β*, IL-6, cAMP, and cGMP ELISA kits (R&D Systems, Minneapolis, MN, USA).

### 2.7. Real-Time Polymerase Chain Reaction (RT-PCR)

PDE2A and NF-*κ*B/p65 mRNA were measured by RT-PCR. Following the manufacturer's protocol, total RNA was isolated from homogenized ipsilateral L4–L6 spinal dorsal horns using the TRIzol reagent (Invitrogen Corp., Carlsbad, CA, USA). Total RNA was then reversely transcribed into cDNA, with the MML-V reverse transcriptase kit (Western Biotechnology, Chongqing, China). PDE2A and NF-*κ*B/p65 mRNA were examined by a PCR system (FTC2000q PCR System; Conrem, Canada) with real-time PCR master mix kit (Fermentas, Glen Burnie, MD, USA). PDE2A and NF-*κ*B/p65 mRNA level were quantified relative to *β*-actin using the relative quantification 2^−∆∆CT^ method.

### 2.8. Western Blotting

The protein expressions of PDE2A and NF-*κ*B/p65 were analyzed by western blotting. Ipsilateral spinal dorsal horn tissue from L4–L6 were homogenized by radio immunoprecipitation assay (RIPA) lysis buffer (Western Biotechnology, Chongqing, China) at 100 mg tissue per mL and centrifuged. Then, the samples were separated on a 10% sodium dodecyl sulfate- (SDS-) polyacrylamide gel electrophoresis (PAGE) gel for 45 minutes and electrophoretically transferred onto a polyvinylidene difluoride (PVDF) membrane (Millipore, USA) for 1 hour at 37°C. The membranes were blocked with 5% fat-free milk in Tris buffer solution (TBS-T) with 0.01% Tween 20 (TBS-T) (Sigma-Aldrich) for 2 hours at 37°C to eliminate nonspecific binding. The membranes were washed in Tris-buffered saline (TBS-T) and incubated with primary PDE2A and NF-*κ*B/p65 antibody (1 : 1000 dilution, Western Biotechnology, Chongqing, China) and rabbit-anti-GAPDH (1 : 3000 dilution, Western Biotechnology, Chongqing, China). The membranes were then incubated at 4°C overnight, washed with TBS-T, and incubated with anti-rabbit secondary antibody (1 : 5000 dilution; Western Biotechnology, Chongqing, China) for 1.5 hours at 37°C. After adequate washing with TBS-T, the protein bands were visualized by enhanced chemiluminescence according to the manufacturer's instructions. Signals were quantified by the UVP gel Imaging System (BioSpectrum, USA), and values were normalized to GAPDH.

### 2.9. Statistical Analysis

SPSS software (SPSS 20.0, Chicago, IL) was used. Data are presented as the mean ± SEM. The data were measured by Student–Newman–Keuls post hoc test for normal distributions, and analyses between groups were performed using one-way analysis of variance (ANOVA). Results were considered statistically significant if the *P* value was less than 0.05.

## 3. Results

### 3.1. 10, 20, or 30 *μ*g/mL O_3_ Treatment Improved the Mechanical PWTs in Chronic Radiculitis Rats

**Figure 1:**
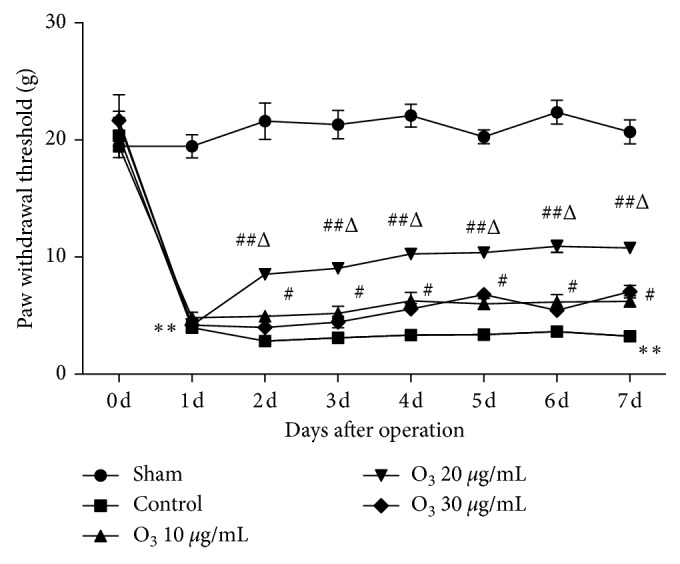
Treatment with low-concentration O_3_ improved mechanical allodynia in chronic radiculitis rats. Implanted autologous nucleus pulposus (NP) led to ipsilateral radicular inflammation. There was no significant difference in paw withdrawal thresholds (PWTs) between groups to mechanical stimuli prior to operation. Ipsilateral PWTs in the control group and 10, 20, and 30 *μ*g/mL O_3_ groups were significantly reduced on day 1 after NP implantation (*P* < 0.05) compared to the PWT before the operation and to the sham group. After intrathecal O_3_ administration, the ipsilateral PWTs were significantly elevated in the 10, 20, and 30 *μ*g/mL O_3_ groups (*P* < 0.05) from day 2 to day 7 after NP implantation. Compared with the 10 *μ*g/mL and 30 *μ*g/mL O_3_ groups, the ipsilateral PWTs were significantly increased in the 20 *μ*g/mL O_3_ group. The values are expressed as mean ± SEM. ^*∗*^*P* < 0.05 vs. sham group; ^#^*P* < 0.05 vs. control group; ^Δ^*P* < 0.05 vs. 10 *μ*g/mL and 30 *μ*g/mL O_3_ groups.

Mechanical PWTs were not significantly different between groups before the operation (*P* > 0.05). There was no significant difference in the sham group PWTs after the operation compared to before the operation (*P* > 0.05). Ipsilateral mechanical PWTs were significantly reduced after operation in the control group (*P* < 0.05) and in the 10, 20, and 30 *μ*g/mL O_3_ groups (*P* < 0.05) compared to the sham group and the within-group measurements prior to operation. Ipsilateral mechanical PWTs were significantly improved in the 10, 20, and 30 *μ*g/mL O_3_ groups from days 2 to 7 after operation (*P* < 0.05) compared to the control group. In addition, mechanical PWTs on days 2–7 were increased significantly in the 20 *μ*g/mL O_3_ group (*P* < 0.05) compared to the 10 or 30 *μ*g/mL O_3_ groups (Figure 1).

### 3.2. 10, 20, or 30 *μ*g/mL O_3_ Reversed the Increased Spinal TNF-*α*, IL-1*β*, and IL-6 Protein Expression Levels in Chronic Radiculitis Rats

**Figure 2:**
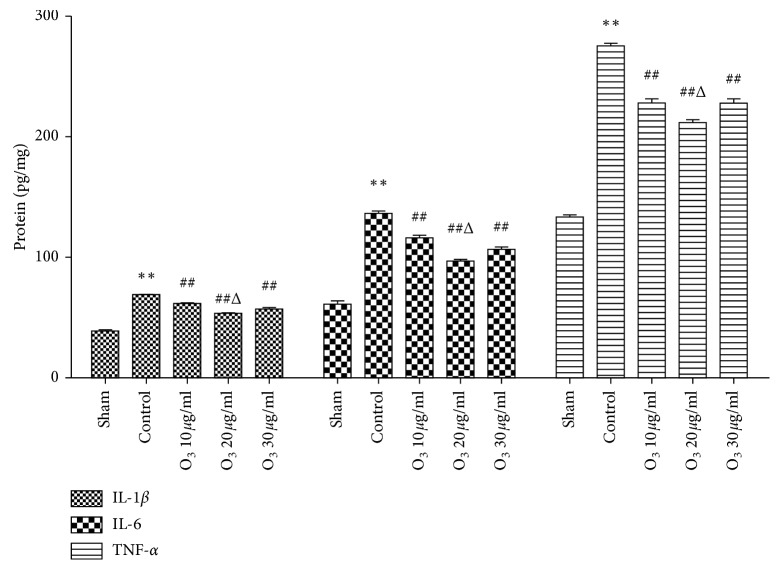
Low-concentration O_3_ treatment reversed the overexpression of tumor necrosis factor- (TNF-) *α*, interleukin- (IL-) 1*β*, and IL-6 in chronic radiculitis rats. TNF-*α*, IL-1*β*, and IL-6 proteins were all significantly increased in the control group (*P* < 0.05) on day 7 compared to the sham group. Compared to the control group, TNF-*α*, IL-1*β*, and IL-6 proteins were significantly decreased in the 10, 20, and 30 *μ*g/mL O_3_ groups (*P* < 0.05). These effects were most noticeable in the 20 *μ*g/mL O_3_ group (*P* < 0.05) compared to those in the other O_3_ groups. The values are expressed as mean ± SEM. ^*∗*^*P* < 0.05 vs. sham group; ^#^*P* < 0.05 vs. control group; ^Δ^*P* < 0.05 vs. 10 *μ*g/mL and 30 *μ*g/mL O_3_ groups.

There were significant increases in spinal TNF-*α*, IL-1*β*, and IL-6 protein expression levels (*P* < 0.05) in the control group compared to the sham group on day 7 after operation. However, the protein expressions of spinal TNF-*α*, IL-1*β*, and IL-6 were significantly lower in the 10, 20, and 30 *μ*g/mL O_3_ groups (*P* < 0.05) compared to the control group. Furthermore, the expression levels of spinal TNF-*α*, IL-1*β*, and IL-6 in the 20 *μ*g/mL O_3_ group were significantly decreased (*P* < 0.05), compared to the 10 or 30 *μ*g/mL O_3_ groups (Figure 2).

### 3.3. Effects of 10, 20, or 30 *μ*g/mL O_3_ on cAMP and cGMP in the Spinal Cord of Chronic Radiculitis Rats

**Figure 3:**
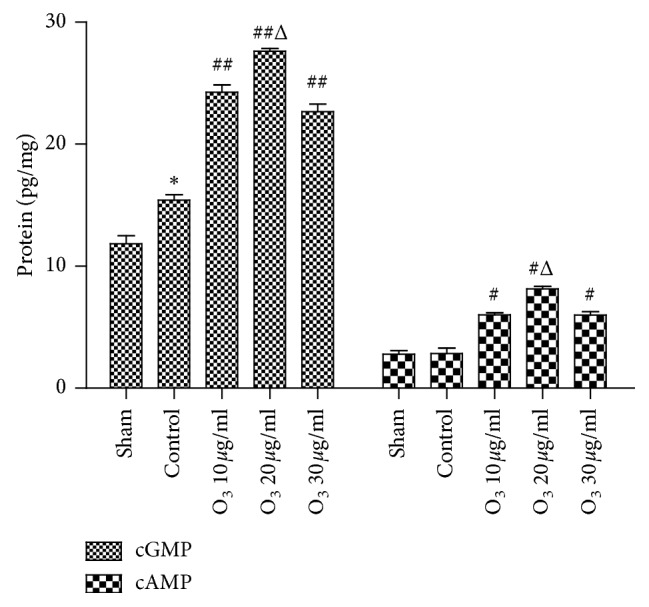
Low-concentration O_3_ upregulated the expressions of cyclic adenosine monophosphate (cAMP) and cyclic guanosine monophosphate (cGMP), but especially cGMP, in chronic radiculitis rats. cGMP was significantly increased in the control group after nucleus pulposus implantation on day 7 (*P* < 0.01). Both cGMP and cAMP were significantly increased to a greater extent in the 10, 20, and 30 *μ*g/mL O_3_ groups (*P* < 0.05) at day 7; this effect was especially pronounced for cGMP. These effects were most noticeable in the 20 *μ*g/mL O_3_ group (*P* < 0.05). The values are expressed as mean ± SEM. ^*∗*^*P* < 0.05 vs. sham group; ^#^*P* < 0.05 vs. vehicle group; ^Δ^*P* < 0.05 vs. O3 10 *μ*g/mL and 30 *μ*g/mL groups.

There was a significant increase in the expression level of cGMP in the control group (*P* < 0.05) compared to the sham group. However, cAMP expression was not significantly different between the control and sham groups (*P* > 0.05). The protein expression levels of both cAMP and cGMP were significantly increased in the 10, 20, and 30 *μ*g/mL O_3_ groups (*P* < 0.05) compared to the control group. Furthermore, the increases of cAMP and cGMP were the most significant in the 20 *μ*g/mL O_3_ group (*P* < 0.05), compared to the 10 or 30 *μ*g/mL O_3_ groups (Figure 3).

### 3.4. 20 *μ*g/mL O_3_ Downregulated the Overexpression of Spinal PDE2A and NF-*κ*B/p65 in Chronic Radiculitis Rats

**Figure 4:**
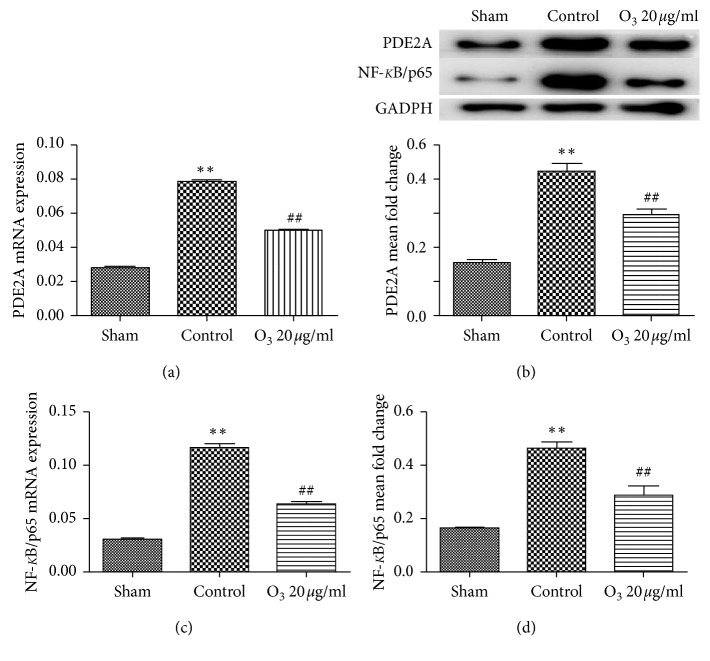
20 *μ*g/mL oxygen/ozone suppressed the expression of phosphodiesterase 2A (PDE2A) and nuclear factor- (NF-) *κ*B/p65 mRNA and protein on day 7 in chronic radiculitis rats. The expression of both PDE2A mRNA (a) and protein (b) in the ipsilateral spinal cord was significantly increased on day 7 in the chronic radiculitis control group (*P* < 0.05). 20 *μ*g/mL O_3_ significantly reversed the overexpression of PDE2A that was induced by autologous nucleus pulposus implantation (*P* < 0.05). The expressions of both NF-*κ*B/p65 mRNA (c) and protein (d) in the spinal cord were also elevated on day 7 in the control group (*P* < 0.05). Both NF-*κ*B/p65 mRNA and protein expressions were significantly decreased in the 20 *μ*g/mL O_3_ group (*P* < 0.05) compared to the control group. The values are expressed as mean ± SEM. ^*∗*^*P* < 0.05 vs. sham group; ^#^*P* < 0.05 vs. control group.

The mRNA and protein expression levels of PDE2A and NF-*κ*B/p65 in the spinal cord on day 7 after operation were significantly upregulated in the control group (*P* < 0.05) compared to the sham group. However, the expression levels of PDE2A and NF-*κ*B/p65 in the 20 *μ*g/mL O_3_ group were significantly downregulated (*P* < 0.05) compared to the control group (Figure 4).

## 4. Discussion

LDH is one of the most familiar diseases that are commonly accompanied by severe radiculitis. Radiculitis causes immense suffering for patients with LDH. Radicular inflammation is one of two common reasons for pain induced by lumbar disc herniation, the other reason being the direct compression of the herniated disc [15, 24, 25]. Many different drugs and minimally invasive techniques, such as nonsteroidal anti-inflammatory drugs (NSAIDs), epidural injection of steroid hormones [26], and/or O_3_, have been used for the treatment of radiculitis in LDH. Among these treatment methods, O_3_ therapy is a common, minimally invasive technique used in some countries, including Italy, China, and Canada, where it has recently been widely applied in the clinical management of pain [24, 25].

Ozone is an unstable gas with a pungent smell, which has a dual role. To date, O_3_ therapy has been widely used in oncology, gynecology, skin and mucosal infections, pain management, and other medical conditions because of its anti-inflammatory, antioxidant, antiseptic, and disinfectant properties [26, 27]. Furthermore, O_3_ treatment has been shown to have therapeutic effects in patients with lumbar disc herniation [25, 28]; Giurazza et al. found that O_3_ therapy used for the treatment of low back pain was safe and effective and that the benefits lasted for up to 10 years after treatment [29]. Melchionda et al. reported that paravertebral injections with oxygen-ozone could induce a direct improvement in radicular inflammation and pain [10]. Bocci found that different concentrations of ozone have different and even contradictory effects [30]. In our previous studies, we also found that intrathecal injection of high concentration ozone (40–60 *μ*g/mL) could induce neurotoxicity, whereas intrathecal injection of low-concentration ozone (<40 *μ*g/mL) is a rarely induced neurotoxicity. [14]. Although O_3_ therapy is now used worldwide, there are very few studies aimed at investigating the mechanisms underlying the use of intrathecal injection of low-concentration O_3_ for the treatment of radiculitis caused by LDH.

TNF-*α*, IL-1*β*, and IL-6 are potent proinflammatory factors, which play an important role in radicular pain [3–5]. Some scholars found that a single subcutaneous injection of O_3_ could decrease the overexpression of IL-1*β* in SNI models [15]. In other studies, O_3_ was found to decrease levels of TNF-*ɑ* and IL-1*β* and also prevent demyelination [31–33]. In the present study, we confirmed that mechanical PWTs decreased significantly during the 7 days following NCLDH operation and that spinal levels of TNF-*α*, IL-1*β*, and IL-6 increased significantly in radiculitis rats. Furthermore, intrathecal injection of 10, 20, or 30 *μ*g/mL O_3_ reduced mechanical allodynia and suppressed the overexpression of TNF-*α*, IL-1*β*, and IL-6 in the spinal cord. We speculate that intrathecal administration of low-concentration O_3_ diminishes radicular inflammation by reducing TNF-*α*, IL-1*β*, and IL-6 in radiculitis rats. We found that intrathecal injection of 20 *μ*g/mL O_3_ led to the most significant reduction of mechanical allodynia and the largest decreases in the spinal TNF-*α*, IL-6, and IL-1*β* levels. These results are consistent with the opinion that ozone with proper concentration could be atoxic and therapeutic [12, 13].

PDEs are enzymes that regulate the physiological function by hydrolyzing cAMP and/or cGMP and are classified into 11 families, PDE1 to PDE11. There is general consensus that PDE2A is abundant in the central nervous system of mammals, especially in the laminae I and II of spinal dorsal horn in rats [20, 34]. In addition, PDE2A might contribute to acute inflammatory pain processing [19]. Recent experiments have detected that an inhibitor of PDE2A had anti-inflammatory and analgesic effects [35]. In a previous study, we found that the expression of PDE2A was upregulated in NCLDH rats, and that the PDE2A inhibitor, BAY 60-7550, could suppress the release of spinal TNF-*ɑ*, IL-6, and IL-1*β* and also improve the mechanical allodynia by increasing the levels of cAMP and cGMP [21]. However, the signaling pathways between PDE2A and low-concentration O_3_ are not clear. In the present study, we found that 20 *μ*g/mL O_3_ could downregulate the overexpression of PDE2A and upregulation of cGMP and cAMP. Therefore, we inferred that low-concentration O_3_ decreased the overexpression of spinal PDE2A, which lessens the hydrolysis of cAMP and cGMP, which leads to alleviation of radiculitis and mechanical allodynia.

Some scholars found that both cAMP and cGMP, as well as nuclear factor-kappa B (NF-*κ*B), are components of downstream signaling pathways of PDEs [11, 20, 26]. Moreover, researchers discovered that inhibition of NF-*κ*B induces increased levels of cGMP [36]. Additionally, cGMP and NF-*κ*B are involved in antioxidative and anti-inflammatory treatments for cancer, neurodegenerative diseases, and low back pain [37–39]. The NF-*κ*B/p65 heterodimer is an important transcription factor in the development of inflammation and pain and is widely expressed in the central nervous system [36]. Luo et al. found that NF-*κ*B/p65 in the spinal cord was activated in chronic arthritic models and that inhibiting the NF-*κ*B/p65 pathway could reduce painful inflammatory disorders [35]. In the current study, we found that the level of NF-*κ*B/p65 in the spinal cord significantly increased after NP implantation; furthermore, 20 *μ*g/mL O_3_ downregulated the expressions of NF-*κ*B/p65 and increased cGMP and cAMP in chronic radiculitis rats. Therefore, we speculate that low-concentration O_3_ inhibits inflammation by modulating the PDE2A-cAMP/cGMP-NF-*κ*B/p65 signaling pathway.

## 5. Conclusion

Intrathecal injection of low-concentration O_3_ (10, 20, or 30 *μ*g/mL) reduces the overexpression of TNF-*ɑ*, IL–6, and IL-1*β* and also significantly alleviates mechanical allodynia in chronic radiculitis rats. Furthermore, 20 *μ*g/mL O_3_ decreases the overexpression of PDE2A and NF-*κ*B/p65 while also upregulates cGMP and cAMP. We believe that radiculitis and mechanical allodynia might be attributed to the PDE2A-cAMP/cGMP-NF-*κ*B/p65 signaling pathway in chronic radiculitis rats and that low-concentration O_3_ attenuates radiculitis and mechanical allodynia by altering this signaling pathway.

### 5.1. Study Limitations

Although 20 *μ*g/mL O_3_ produced the strongest anti-inflammatory effects in the present study, we cannot infer that 20 *μ*g/mL O_3_ is the best concentration to use in the treatment of radiculitis patients. However, our results indicate a potential clinical application of low-concentration O_3_ in the treatment of patients with radiculitis from LDH.

## Data Availability

The data used to support the findings of this study are available from the corresponding author upon request.
